# Introduction to Special Issue on Advancing Health Equity Among Black Communities

**DOI:** 10.1007/s11121-023-01622-1

**Published:** 2023-11-30

**Authors:** Katrina J. Debnam, Caryn R. R. Rodgers, Paula Smith

**Affiliations:** 1 University of Virginia, Charlottesville, USA; 2 Albert Einstein College of Medicine, Bronx, USA; 3 University of Utah, Salt Lake City, USA

**Keywords:** African American, Black, Health equity, Racism

## Abstract

The current paper serves as an introduction to this special issue, Advancing Health Equity among Black Communities, in which we provide an overview of the papers included. Specifically, we summarize the papers covered in the special issue and highlight some of the common themes. The impetus for this special issue originated from a culmination of the COVID-19 pandemic, continued murders of Black people by police officers, and an unsettling political climate (e.g., Galea & Abdalla, 2020). While the impact of individual racism has been studied extensively, the insidious and pervasive impact of structural racism is less understood. Structural racism is a system in which embedded values, practices and policies facilitate and perpetrate the continued differential treatment of people based on race and becomes an almost hidden influence on the way an institution functions. For this special issue, prevention scientists were invited to submit conceptual and empirical research reflecting their understandings of structural racism as it operates in U.S. systems (e.g., education, justice, housing, workforce) and contributes to health inequities in the lives of Black Americans. The submissions also demonstrate how prevention scientists can leverage translational science to impact policies, practices, and procedures to promote equitable and sustainable change for Black communities.

The impetus for this special issue, Advancing Health Equity among Black Communities, originated from a culmination of the COVID-19 pandemic, continued murders of Black people by police officers, and an unsettling political climate (Galea & Abdalla, 2020; Laurencin & McClinton, 2020; Maness et al., 2021). These events amplified the ways in which Black people in the United States (U.S.) are not able to access the same healthcare, justice, educational expectations, housing, economic benefits, and life expectancy as white people living in the US. During the immediate aftermath of these events, there were calls and movements focusing on ways to create equity and optimal health and experiences for Black people in the U.S. There were many calls to action by for profit and not for profit companies. Companies were providing free services to Black people, entertainment apps and networks were featuring Black television, movies, and art, and companies were consciously making sincere efforts in recognizing the needs of their Black employees and a major push for Diversity, Equity and Inclusion was birthed (e.g., Salles et al., 2021; Sobo et al., 2020). Approximately 3 years after the onset of the pandemic, many of these efforts no longer exist with the same vigor and visibility, but the impact of structural racism continues to create an environment where Black people in the U.S. have disparate experiences that negatively impact many aspects of their daily life.

While the impact of individual racism has been studied extensively, the insidious and pervasive impact of structural racism is less understood. Structural racism is a system in which embedded values, practices and policies facilitate and perpetrate the continued differential treatment of people based on race and becomes an almost hidden component of the way an institution functions. These institutional practices and norms exist in reinforcing activities that at times are attributed to an individual or groups effort but not recognized as an intrinsic aspect of a societal, cultural and/or institutional norms or way of operating (Braveman et al., 2022). Because of the ways in which multiple systems and institutions enact racist policies and practices, there are numerous hypothesized pathways between structural racism and health outcomes among Black Americans (Bailey et al., 2017). For the purposes of this special issue, we have foregrounded our understanding of structural racism using the work of Bailey and her colleagues (2017). Specifically, they define structural racism as “the totality of ways in which societies foster racial discrimination through mutually reinforcing systems of housing, education, employment, earnings, benefits, credit, media, health care, and criminal justice. These patterns and practices in turn reinforce discriminatory beliefs, values, and distribution of resources.” (Bailey et al., 2017, p. 1453). While the majority of these policies and practices no longer explicitly name race (e.g., Jim Crow laws which required medical facilities to be racially segregated resulting in inadequate health care), the lasting impact of and cost of these practices in U.S. systems and structures are ongoing (e.g., inadequate access to health insurance and poorly resourced health-care facilities in racially segregated communities) and greatly contribute to the health and wellbeing of Black Americans.

The primary objective for this special issue was to illuminate the importance of prevention science as an agent of health equity for Black people living in the U.S. The U.S. Department of Health, Healthy People 2030 initiative defines health equity as “the attainment of the highest level of health for all people” (Office of Disease Prevention and Health Promotion, n.d.). The initiative further states that “achieving health equity requires valuing everyone equally with focused and ongoing societal efforts to address avoidable inequalities, historical and contemporary injustices, and social determinants of health — and to eliminate disparities in health and health care” (U.S. Department of Health & Human Services, 2022, p. 9). The focus of this work is to explore ways in which we, as prevention scientists, can contribute by developing initiatives and work aimed at equity for Black people in the U.S. The field of prevention science is still relatively young (25+ years old) and continues to draw on diverse disciplines, including sociology, psychology, public health, among others. While individual interventions continue to be a critical component of the prevention research cycle, prevention science needs to continue to push forward with multi-level interventions and interventions to effect change at a systems level.

For this special issue, prevention scientists were invited to submit conceptual and empirical research reflecting their understandings of structural racism as it operates in U.S. systems (e.g., education, justice, housing, workforce) and contributes to health inequities in the lives of Black Americans. The authors were also asked to discuss how prevention scientists leverage translational science to impact policies, practices, and procedures to promote equitable and sustainable change for Black communities. The articles presented in this special issue include a range of topics, from academic achievement to doula care, using a variety of methodological approaches including qualitative, quantitative, and secondary data analysis. Taken together, the articles further our understanding of health inequities in Black communities and provide guidance for developing preventive interventions that prioritize outcomes of optimal health for Black people living in the U.S.

The first section of this special issue presents conceptual frameworks for advancing the field of prevention science to promote health equity. First, an article by Murry and colleagues (in press) offers prevention scientists concrete research priorities and steps that hold promise for promoting health equity by addressing systemic racism. The insights from Murry and colleagues were born out of a 2-year presentation series on systemic racism sponsored by the NIH-funded Prevention Science and Methodology Group. Of note, the authors call for a change from individual or “microlevel” interventions to interventions that address structural barriers which create and maintain inequities and injustices (Murry et al., in press). This call echoes an article commissioned by the Society for Prevention Research (SPR), published in a previous issue of Prevention Science that introduces the use of an *Ecosystemic Framework* for promoting health equity that extends Bronfrenbrenner’s classic ecological framework to include pathways of stagewise effects and drivers of equity outcomes (Boyd et al., 2023). In addition, both articles stress the need for a retooling of methods and measures in prevention science to evaluate contextual impacts on interventions and intervention-related equity outcomes. Next, Woods-Jaeger et al. (in press) describe the Youth Empowering Advocating for Health (YEAH) strategy for translational research with Black youth. Building on the principles of youth participatory action research, the YEAH strategy uses photovoice and advocacy training sessions to identify community concerns and priorities. In this article, Woods-Jaeger and her colleagues provide the historical origins and the development of YEAH, describes its features and activities, and reflects on key lessons learned from implementation in three Black communities.

The next section of articles in this special issue focuses on protective factors unique to Black communities that could be harnessed to reduce health inequities. A study by Summers-Gabr and colleagues (in press) examines emotional regulation and prosocial development. Data from a sample of Black youth demonstrates that not only is racial discrimination associated with anxiety/depression and suicidal thoughts, but it also impacts protective factors like emotional regulation and prosocial behavior development that could serve to mitigate experiences of discrimination.

The following two papers examine the role of racial socialization as a protective factor for Black youth. Racial socialization is defined as the verbal and nonverbal racial communication between families and youth (Hughes et al., 2006) and has, in previous research, been identified as a protective factor to a host of academic and emotional well-being outcomes for youth (Lesane-Brown, 2006; Neblett Jr et al., 2012; Umaña‐Taylor & Hill, 2020). The study led by Berkel (in press) in this special issue examines the effect of the Strong African American Families (SAAF) program, a culturally tailored prevention intervention for rural African American families, on preventing maladaptive coping among African American youth in response to discrimination. Previous studies of SAAF have shown significant impacts on youth assets, reductions in conduct problems, substance use, and early sexual activity, but this is the first study to test the ways in which it protects against the negative consequences of experiencing racial discrimination. In this study, the authors used mediation analyses to determine if racial socialization and Black pride would mitigate the effects of discrimination on psychological functioning and risky behaviors. Their findings revealed significant indirect effects on psychological functioning and risky behaviors but a nonsignificant interaction between Black pride and discrimination. The authors discuss the importance of Black pride and racial socialization as protective factors for African American youth regardless of exposure to discrimination but also encourage additional research to prevent exposure to discrimination for African American adolescents.

Similarly, Lambert et al. (in press) examined the associations between ethnic-racial socialization messages and school engagement and achievement among a sample of Caribbean Black and African American youth. Their results similarly showed that the benefits of ethnic-racial socialization messages and frequency of communication about race were not sufficient to combat the effects of teacher discrimination. These findings further establish the importance of racial socialization as a protective factor for Black youth.

An original article by Dinizulu and colleagues (in press) introduced a social justice service-learning intervention for Black youth provided in a faith-based summer camp. Adapted by a community advisory board, the Stimulating Maturity through Accelerated Readiness Training (SMART) curriculum was delivered by church staff to middle school aged youth. Study findings demonstrated the feasibility, acceptability, and promise of the service-learning intervention to improve psychological engagement and academic motivation. Furthermore, the authors emphasized the importance of Black church in promoting civic engagement and mental health among Black youth. Lastly, the article by Barbarin et al. (in press) considered how the development of social competencies co-exists with problem behaviors during adolescence. Using data from the National Health Interview Study collected by the US Census Bureau for the Center for Disease and Control, Barbarin and colleagues created profiles of African American youth based on family indicators of adversity, reports of affability, altruism, and conduct problems. These profiles are examined in relationship to their psychological and physical health outcomes. Results suggested that while prosocial competencies can co-occur with conduct problems, multiple family stressors hamper social competency development.

The concluding articles in this special issue consider how prevention science research can be used to change policies and procedures that are negatively impacting Black communities. Roman (in press) brings together research on help-seeking, social determinants of health, discrimination in criminal justice system functioning, and victim services, and synthesizes it into a conceptual model that details the factors that shape decision-making regarding reporting victimization to police and subsequent help-seeking among Black Americans. Roman describes how her findings from data collected from 91 victims of community violence can be used to inform and guide policy. For example, pairing hospital violence intervention programs with community health workers who have lived experiences with violence could create more sustained engagement in services by victims. Finally, Temple and Varshney (in press), provided an economic framework that prevention scientists could use to persuade policymakers to grow funding for doula care and make sustainable investments in maternal health. This data-driven philanthropy approach can be applied to other issues to expand culturally informed services provided to Black communities.

One main goal of this special issue was to draw attention to the multitude of ways that prevention science methods, interventions, and policy research can be leveraged to address the structural and individual racism that exacerbates health inequities for Black people living in the U.S. (e.g., Boyd et al., 2023). Specifically, prevention science can inform epidemiological, etiological, interventions (from efficacy to effectiveness), and dissemination including scientifically informed guidance for policy makers and health care administrators to ensure that inequities in health policy and procedures are addressed. Advances in prevention science can also be brought to bear to reimagine methods, measurement, translational, and ethical community engagement to promote health equity for Black and other minoritized communities. Furthermore, prevention science provides an evidence-informed understanding of protective factors that may offer additional resiliency for Black communities whose lives are cut short due, in part, to health inequities stemming from a historical legacy of anti-Black racism. Lastly, prevention research can support those seeking to make changes in the policies and practices that sustain inequities in various sectors of U.S. society, including health care, law enforcement, housing, and education, among others.

Our hope in presenting this special issue is to raise awareness and draw attention to the ways in which anti-Black sentiment continues to impact the lives of everyday communities and offer hope for reducing the impact of structurally embedded and seemingly intractable social determinants of health. Our call to action for the prevention science community is to extend their research to uncover pathways to decrease the risks associated with structural- and individual-levels of racism and to conduct research that increases our understanding of protective factors that may enable disparities to be lessened or at the very least, the impact of the disparities to be lessened. Specifically, Barbarin and his colleagues recommend that more research be conducted to assess the efficacy and effectiveness of prevention programming incorporating positive youth development approaches when working with Black youth in their schools, communities, and families. They share that this focus on the prosocial strengths of Black youth and their commitment to serve others is key in neutralizing the negative effects of racism and discrimination. These recommendations are echoed by others in this issue including Dinizulu and her colleagues who encourage prevention researchers to consider the inclusion of critical service learning in our interventions with Black youth to raise awareness of and action against racist practices; Woods-Jaeger and her colleagues push the field of prevention to consider the ways that investigators should engage with Black youth to address aspects of structural racism; and Berkel and her colleagues inspire us to begin research that identifies the ways in which families interventions can help improve poor mental health among Black youth that occurs as a result of discrimination and call for additional research to reduce Black youth exposure to discrimination — a call that is echoed by Summers Gabr and her colleagues in this issue.

Furthermore, we hope that this research will underscore the importance of developing policy solutions that ameliorate the harms of health inequities, from those policies that intentionally harm minoritized communities as well as those that unintentionally cause harm. One example, taken from this set of papers is the work of Temple and Varshney (in press) who encourages prevention scientists to consider how research can inform policy to advocate for health care solutions that may ameliorate biases in the health care system. While their example is doula care for Black women giving birth, their model can be applied across the health care spectrum to provide more equitable care and thus reduce long standing health inequities that Black people face. Similarly, Roman provides a model of policy change that helps us to begin critical policy conversations that can better serve the Black community, including those victims of violence who may not seek help from a law enforcement system that they perceive as biased or untrustworthy.

In addition to policy changes, prevention science can also uncover the mechanisms of bias and discrimination that undergird everyday practices which serve to preserve and deepen the inequities in our human service, health care, law enforcement, and educational systems. As is clear from these set of papers, there is clearly more work to be done in practice settings where the unchecked bias (intentional or unrecognized) of those who work with Black communities continues to have a negative impact. Lambert and her colleagues provide empirical evidence that discriminatory behavior of teachers is experienced as harmful by Black youth. This finding points to a clear and urgent need for anti-racist professional development training to occur at the pre-service and in-service levels of our nation’s teacher education programs. Murry and her colleagues underscore this by sharing three recommendations for our collective work in prevention research. First, they share that we need research that extends beyond individual level interventions to include the ecological communities and systems with which the individual engages. Next, they call for an expansion of prevention research to include real-life complexity so that we can better understand the root societal and systemic nature of discrimination and racism that impacts Black people on a daily basis. Lastly, Murry and her colleagues implore us to examine how our own institutions and research continue and sustain racist practices by not engaging Black communities that we research in, by hoarding resources that could be shared with Black communities, and by not acknowledging the many strengths and resilience that have allowed Black people to survive 400-plus years of subjugation, enslavement, and hostility.

As we approach the tipping point for population-level shifts in the U.S., we must be ever vigilant to ensure that the growing hateful racist rhetoric voiced by a minority does not enable the unraveling of basic human rights and thus deepen the current health disparities. We have passed the moment of racial reckoning that characterized the early part of the 2020s, but the efforts for health equity are now even more critical as we have seen recent attacks on research and programs aimed at reducing health inequities. These attacks have increasingly focused on creating a narrative of fear and they have exacerbated anti-Black and other xenophobic sentiments. The urgency for this work remains as unarmed Black men (and to a lesser extent Black women) continue to be killed at alarming rates by law enforcement (e.g., Bunn, 2022), continue to suffer in health care systems where their particular biological profiles are not well understood (e.g., Rosa et al., 2022), and continue to be funneled from educational systems into the juvenile and criminal justice systems at exceedingly high rates (e.g., Keyes, 2022).
